# Physiotherapist Online Assessment in Patients with Stroke: Protocol for a Systematic Review and Meta-Analysis

**DOI:** 10.3390/jcm14072311

**Published:** 2025-03-28

**Authors:** María-José Estebanez-Pérez, Pablo Pastora-Estebanez, Ismael Romero-García, Maria Jesus Vinolo-Gil, Rocío Fernández-Navarro, José-Manuel Pastora-Bernal

**Affiliations:** 1 Department of Physiotherapy, Faculty of Health Sciences, University of Granada, 18016 Granada, Spain; 2 Department of Economy, Faculty of Economics and Business Studies, University of Málaga, 29013 Málaga, Spain; pablopastora@uma.es; 3 Department of Physiotherapy, San Isidoro University Centre, Pablo de Olavide University, 41013 Sevilla, Spain; iromero@centrosanisidoro.es; 4 Department of Nursing and Physiotherapy, Faculty of Nursing and Physiotherapy, University of Cadiz, 11009 Cadiz, Spain; mariajesus.vinolo@uca.es; 5 Department of Physiotherapy, Comarcal Hospital of Melilla, Ingesa Melilla, 52005 Melilla, Spain; rociofernava@ugr.es; 6 Department of Physiotherapy, Faculty of Health Sciences, University of Málaga, 29071 Málaga, Spain; jmpastora@uma.es

**Keywords:** tele-assessment, systematic review, telerehabilitation, physiotherapy, stroke

## Abstract

**Background**: About 15 million people suffer a stroke each year, of which 10–15% occur in people under 50 years of age. The clinical management of neurological disorders depends on reliable diagnostic tools to identify impairments and aid in the early and accurate detection of disease. The objective of this study is to present a systematic review protocol for identifying the scientific evidence on the use of tele-assessment compared with in-person assessment delivery by physiotherapists for stroke patients. This protocol was registered on the International Prospective Register of Systematic Reviews (PROSPERO) database (CRD42024613552). **Methods**: Original studies of any design in which physiotherapy tele-assessment using videoconferencing compared with face-to-face assessment for patients with stroke conditions will be included. The research will be carried out in PubMed/Medline, Cochrane Library, PEDro (Physiotherapy Evidence Database), and NICE. The risk of bias will be assessed using the Quality Appraisal Tool for studies of diagnostic Reliability (QAREL) and the Quality Assessment of Diagnostic Accuracy Studies (QUADAS). **Results**: The screening, selection, and analysis process will be conducted by two independent researchers and reviewed by a third evaluator to resolve any potential disagreements. The feasibility of conducting a meta-analysis for quantitative data will be evaluated based on the homogeneity analysis of the selected studies. **Conclusions**: We hope that this systematic review protocol will provide scientific evidence for tele-assessment as a physiotherapeutic assessment strategy for stroke patients and that it will be available as a complementary tool to face-to-face physiotherapeutic assessments for specific situations.

## 1. Introduction

According to the World Health Organization (WHO), stroke is “a clinical syndrome consisting of signs of rapidly developing focal neurological disturbances of brain function, lasting more than 24 h or leading to death, with no apparent cause other than vascular disease” [1]. Strokes are usually acute events caused primarily by a blockage [2], or by a sudden hemorrhage [3], that impedes blood flow to the heart or brain. As the second leading cause of death, and the third leading cause of disability, according to the WHO, stroke is considered a major global health concern [4]. The clinical management of neurological disorders depends on reliable diagnostic tools to identify impairments and aid in the early and accurate detection of disease [5]. New assessment tools are required to respond to the needs of the increasingly digitalized or online interaction with patients.

The terms “digital physiotherapy practice” and “telerehabilitation” refer to healthcare services, support, and information delivered remotely through digital communication and devices. The goal of digital physical therapy practice is to enhance the efficiency of physical therapy services by improving access to care and information while optimizing healthcare resources [6]. Recent studies on digital physiotherapy practice and stroke patients have identified a wide variety of tools, technologies, and approaches that can be used for rehabilitation [7]. This requires specific support tools, such as a computer system that provides decision-making support throughout the healthcare process [8]. The college of physiotherapists of Ontario (Canada) emphasizes that all aspects of patient care—including the patient interview, physical assessment, and diagnosis, treatment, maintenance activities, consultation, education, and training—are part of digital physiotherapy practice or telerehabilitation. This may include the use of media such as videoconferencing, email, apps, web-based communication, and wearable technology [9]. A systematic review of the literature is key to validating the effectiveness of digital physiotherapy in stroke patients.

Recent studies have validated digital physiotherapy practice as a suitable alternative [10] or complementary to standard rehabilitation care for stroke patients. This therapy is considered complementary in the face of skepticism from professionals and patients [11]. Scholars have observed that the physical assessment of patients is performed face-to-face, while digital physiotherapy sessions are conducted through digital means [12,13]. Thus, online physical assessment remains an under-explored area.

Online assessment or tele-assessment is a term to describe a relatively new assessment approach that requires special considerations. Most physical evaluation techniques, in physical therapy, have been designed for in-person administration, with scoring norms based on the examinee taking the test face-to-face with the evaluator [14]. Preliminary research indicates that many of these measures yield similar results when conducted via tele-assessment, with the evaluator in a different location [15]. However, the degree of equivalence varies depending on the measure, and research remains inconclusive [16]. Consequently, determining the validity of measures administered through tele-assessment is a challenge [6]. Physical assessment and evaluation with digital assessment tools build on a specific virtual interaction that requires the administration of outcome measures by the patient under the guidance of the physiotherapist [14], demanding a series of recommendations and suggestions [17].

Numerous potential benefits of tele-assessment have been described throughout the literature [18], such as being able to expand service delivery options when in-person care is challenging [19] or potential improvement in the timeliness of service delivery and relevant contextual information gained by performing the examination in the patient’s home environment [20]. Yet, as noticed in a report on digital practice by the World Confederation for Physical Therapy (WCPT) and in the practice guidelines of the American Physical Therapy Association (APTA), digital physiotherapy may imply risks such as potential implementation costs and detriments compared to in-person examination for certain health conditions [18].

In general, the tele-assessment of musculoskeletal conditions by a physiotherapist is consistent but limited in terms of scientific evidence [18]. An example of this is that service users need to assume a more active role in the examination process. They may need to self-palpate during the assessment, caregiver assistance may be required to ensure patient safety, or the self-environment may need to be adapted to the test [21]. The validity of remotely performed assessment compared with in-person assessments has been demonstrated in small trials for some conditions: total knee arthroplasty [22], adults with low back pain [23], elbow disorders [24], and non-articular lower limb pain [25], to name but a few.

Research into the online assessment of musculoskeletal conditions showed substantial to near-perfect agreement between tele-assessment and in-person physical examination findings, suggesting reasonable applicability across diverse patient demographics [20]. These studies have been conducted by the same investigators in laboratory or clinical settings rather than in real-world settings. Therefore, it is uncertain whether the high level of agreement observed in experimental settings would be replicated in real-world scenarios [26].

Regarding the different assessments that a physiotherapist performs in a clinical situation, APTA’s most recent clinical practice guidelines state that physiotherapists may use the results to inform the diagnosis with comparable accuracy to an in-person visit, with low evidence quality and weak recommendation strength [18]. Nevertheless, with respect to physiotherapy tele-assessment in stroke, we were unable to identify any literature reviews that specifically addressed its evidence in comparison to face-to-face physiotherapy assessment. Unresolved questions and paradoxes persist, requiring further clarification.

Future research recommendations by clinical guidelines and reviews of research that can provide evidence for the use of tele-assessment and its clinical effectiveness are amply justified, especially considering the low quality of evidence and weak strength of recommendation in tele-assessment for different musculoskeletal conditions, or the absence of evidence in tele-assessment for conditions such as neurological, pediatric, oncological, cardiovascular, and respiratory.

Our principal objective is to present a systematic review protocol for identifying the scientific evidence on the use of online physiotherapy assessment via videoconferencing compared with in-person physiotherapy assessment delivery for stroke patients. This systematic review protocol aims to answer the following questions: “Is physiotherapy tele-assessment for stroke patients comparable to face-to-face assessment?”, and, “Can tele-assessment for stroke patients be considered a complementary tool to face-to-face physiotherapy assessments?”.

## 2. Methods

To guarantee the comprehensive reporting and execution of this systematic review protocol, the Preferred Reporting Items for Systematic Reviews and Meta-Analyses Protocols (PRISMA-P) guidelines [27] (see Supplementary File S1. Checklist Protocol Review) and recommendations for Systematic Reviews in Telerehabilitation [28] were followed. The final paper will be developed following PRISMA [29] and the Cochrane Handbook for Systematic Reviews of Interventions [30]. The review methodology was preregistered on the International Prospective Register of Systematic Reviews (PROSPERO) with registration number CRD42024613552.

### 2.1. Eligibility Criteria

Primary eligibility criteria required published research measuring the validity and/or reliability (inter-rater and/or intra-rater) of physiotherapy assessment for stroke. The eligibility criteria follow the “PICO” (population, index test, comparator test, and outcome) principle as indicated below.

### 2.2. Inclusion Criteria

#### 2.2.1. Population

The population must include adults [≥18 years] with stroke conditions, ICD-10 (I-60-I-69) [31], and access to a digital device with internet connectivity (smartphone, tablet, or computer); patients diagnosed with a stroke based on clinical symptoms and with stable physical condition who possess pathological and imaging evidence; patients with an assistant and stable physical condition and without cognitive or communication problems.

#### 2.2.2. Index Test

Synchronous tele-assessment (understood as real-time online evaluation via videoconferencing) must be undertaken using a videoconferencing platform with the assessor, or physiotherapist, and participants in remote locations and connected via the internet.

#### 2.2.3. Comparator Test

Tele-assessment must be compared with usual face-to-face assessment processes.

#### 2.2.4. Primary Outcomes

Validity covers inter-rater and intra-rater reliability, accuracy, minimal detectable change score, and measurement error study for physiotherapy assessment components. We define validity as the level of agreement between remote and face-to-face assessments, regardless of the terminology used in the included items. Each physiotherapist will assess face-to-face and/or online the outcome measures of standard clinical tests commonly used in post-stroke rehabilitation management [22].

Reliability refers to the consistency between two or more observations of the same entity. Assessors should be qualified physiotherapists with experience in stroke rehabilitation [32].

Inter-rater reliability measures the agreement between two or more assessors evaluating the same entity, while intra-rater reliability assesses the consistency of repeated observations by a single evaluator. Accuracy refers to the proximity of the final result to the correct or accepted value. The accuracy of physical tele-assessment in the stroke patient will be assessed by comparing the results of the different tests or scales used, in-person and by video call [33].

Minimum detectable change score incorporates and emphasizes the patients’ perspective on treatments and their health statuses, and includes them in the decision-making process. These scores will help determine that the smallest measurable change is not attributable to error. Measurement error study encompasses possible biases [34].

#### 2.2.5. Secondary Outcomes

Secondary outcomes may include sensorimotor impairment, balance, mobility, functional capacity, gait, motor function, grasp, grip, pinch, gross, and any other outcomes not consider as primary clinical variables by the authors.

### 2.3. Exclusion Criteria

Patients who report any medical conditions that may preclude a safe examination, such as hearing or visual impairments, will be excluded to guarantee adequate participation in the tele-assessment. Patients lacking the ability to mobilize independently or requiring the use of an interpreter will be excluded.

We will disregard tele-assessment interventions for monitoring symptoms via sensors, video recorder assessment, automatic app assessments, or physiological parameters only. Studies where the comparison group received no face-to-face usual assessment process, or other remote assessment, and studies where patient auto-reporter assessments have been developed via the internet or another medium will also be excluded.

## 3. Study Design

The search will be carried out in the following databases: PubMed/Medline, Cochrane Library, PEDro (Physiotherapy Evidence Database), and NICE. A free combination of Medical Subject Heading (MeSH) and Keywords with Boolean logic (AND) will be used (see Supplementary File S2. Query search). Publication date or language restrictions will be omitted from the searches. The same search terms will be used in each electronic database. To ensure the scope of this review, bibliographies will be searched manually from the reference list of articles obtained from the electronic search. In addition, we will search the ClinicalTrials.gov registry website for ongoing and unpublished trials. Unpublished data or preprints, if available, will be requested from the corresponding authors.

## 4. Selection Process

**Figure 1:**
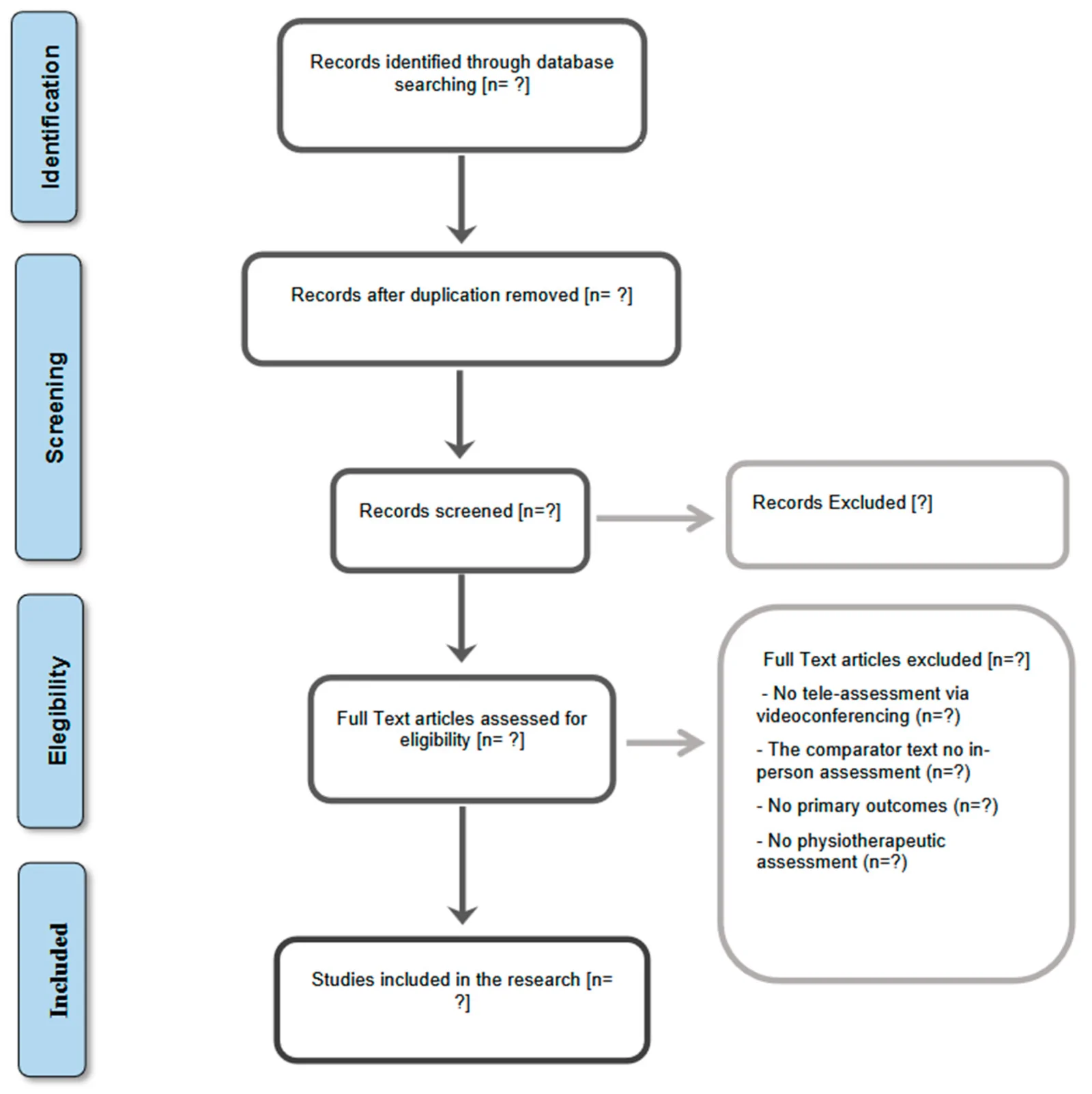
Flow diagram.

Two reviewers will participate in the selection and screening procedure of the accessible literature from the databases. Each reviewer will work independently, without knowledge of the other’s decision. First, the reviewers will apply the inclusion and exclusion criteria when searching the databases by reading the titles and abstracts. Next, the reviewers will apply the eligibility criteria after a full reading of the articles selected in the initial phase. Then, they will assess the methodological quality and risk of bias of the articles included in the previous step and perform a quantitative synthesis of the data of the selected articles. The selected literature will be reviewed by a third independent reviewer to avoid possible disagreements. All researchers will review the full text of all eligible studies. Ultimately, the selected articles will be included in the systematic review. The different phases of the selection process will be documented using a Preferred Reporting Items for Systematic Reviews and Meta-Analyses Protocols (PRISMA-P) diagram [29], as shown Figure 1.

## 5. Data Extraction

A standard form will be created by the research team in order to extract specific data from an article. We will consider the following data:

1. Research characteristics: Title, first author, publication year, study setting, study design, sample size, and diagnostic criteria for stroke.
2. Population characteristics: Age range, gender/sex, comorbidity, cognition, and mobility.
3. Intervention characteristics: Intervention type, delivery devices, types of platforms, mode of delivery (synchronous/asynchronous), location, duration, and results.
4. Comparator intervention: usual face-to-face assessment process, location, duration, and results.
5. Outcome characteristics: Validity, inter-rater and intra-rater reliability, accuracy, minimal detectable change score, and measurement error study for physiotherapy assessment components. As secondary outcomes: sensorimotor impairment, balance, mobility, functional capacity, gait, motor function, grasp, grip, pinch, gross, and any other outcomes that the authors do not consider as primary clinical variables.
6. Analysis method: Statistical methods used, quantitative synthesis, risk of bias, and quality assessment.

Two researchers will pilot the form before its official implementation. Any disagreement will be resolved through discussion and review of the article, with a third researcher being consulted if necessary. Some examples of data extraction are available in Table 1. We intend to complete the data extraction process by the end of December 2024.

## 6. Methodological Quality Check

Two researchers will independently evaluate the risk of bias using the Quality Appraisal Tool for studies of diagnostic Reliability (QAREL) [35] and the Quality Assessment of Diagnostic Accuracy Studies (QUADAS) [36] in order to assess the methodological reliability and validity of the studies.

QAREL has been demonstrated to be a reliable assessment tool for diagnostic reliability studies [20]. Each QAREL item may be answered with “yes”, “no”, or “unclear”. A “yes” response is indicative of good survey quality, while a “no” or “unclear” response indicates an aspect of poor quality. A study’s quality will be classified based on QAREL scores: 67% or more positive responses indicate high quality; 50–66% indicates moderate quality; and less than 50% indicates low quality [35].

Since 2004, the QUADAS tool has provided researchers with an information framework that has improved the quality of diagnostic studies [37], and served as a valid instrument of assessment of the methodological quality of validity studies. If applied consistently, this can facilitate comparisons between studies. The QUADAS tool includes 14 items that can be answered with “yes”, “no”, or “unclear”, where “yes” is considered a good response [36]. A validity study is considered to be of high quality when obtaining a QUADAS score of ≥60% “yes” response [20].

## 7. Statistical Analysis

A narrative approach will be used to summarize the evidence for tele-assessment as a physiotherapeutic assessment strategy for stroke patients, which will be analyzed separately. If sufficient data are available, the following subgroup analyses will be carried out: specific details of participants (time frame of stroke and degree of neurological impairment), types of tele-assessment (platforms, devices, Supplementary Materials, etc.), and research setting (family participation, duration, prior instructions, etc.).

If the studies are methodologically homogeneous, a meta-analysis will be conducted. Review Manager (RevMan V5.3.3) [38] software will be used for the data analysis. Heterogeneity between study results will be assessed by performing a standard chi-square test with a significance level of 0.05. The I2 statistic, which is a quantitative measure of inconsistency between studies, will be calculated to assess heterogeneity. A value of 0% is indicative of homogeneity, while values of 50% indicate a moderate level, and 75% or higher a substantial level of heterogeneity [26]. A fixed-effects model will be applied when no heterogeneity is detected, whereas a random-effects model will be used otherwise.

The intention is to use funnel plots to assess the presence of possible publication biases. Linear regression will be performed to assess funnel plot asymmetry [39]. A narrative summary will be performed if studies are heterogeneous.

## 8. Certainty of Evidence

The Grading of Recommendations, Assessment, Development and Evaluations (GRADE) [34] approach will be used to determine the validity and reliability of digital assessment and clinical management decisions in the included studies. The process involves assessing the quality of evidence based on factors such as risk of bias, inconsistency, indirectness, imprecision, and publication bias [40]. In the case that multiple outcomes are given, a prioritization approach will be used [41]. On the one hand, the certainty of evidence will be rated as high for appropriately designed diagnostic studies in patients with diagnostic uncertainty and direct comparisons of test results with an appropriate reference. On the other hand, the certainty of evidence will be rated as low if uncertainty in any of the domains occurs, as recommended in the GRADE guidelines [34].

## 9. Ethics and Dissemination

Ethical approval and human consent are not required because only secondary data will be used. Modifications and changes will be explained in the final report of this review and will be updated on the Prospective International Register of Systematic Reviews (PROSPERO) website. The results obtained will be disseminated in high-impact journals and at congresses and conferences.

## 10. Discussion

The purpose of this investigation is to carry out systematic reviews and meta-analyses to evaluate the evidence on the use of online physiotherapy assessment via videoconferencing compared with in-person physiotherapy assessment delivery for stroke patients. About 15 million people suffer a stroke each year, of which 10–15% occur in people under 50 years of age (young adult stroke) [42]. Health strategies for stroke management vary worldwide, and it will be interesting to learn about new initiatives that facilitate access to health interventions with scientific evidence in order to enhance their application [43]. Digital health aims to contribute to the maintenance of a high level of health in the population and to strengthen health systems through the transformative capacity of digital technologies for individuals, health professionals, health service provider organizations, and other related agents [44]. We aim to finalize the data synthesis process in the coming months. The final review report will be prepared in accordance with PRISMA standards after integrating and categorizing the data as shown below.

## 11. Conclusions, Strengths, and Limitations

⇒ This research will be the first systematic review on the use of digital physiotherapy assessment compared to face-to-face assessment in adults with stroke.
⇒ This review seeks to determine the scientific evidence on the use of a digital assessment in a study of populations with a high prevalence.
⇒ The results may provide answers to questions and paradoxes that currently remain unresolved.
⇒ This review will adhere to the Preferred Reporting Items for Systematic Review and Meta-Analyses in order to reduce bias.
⇒ There may be high heterogeneity in the selected studies due to different diagnostic criteria, types of devices used, and characteristics of adults with stroke.

## Figures and Tables

**Table 1:** Example of data extraction process.

Data to Be Extracted	Item
Publication ID	Title, first author, and publication characteristics (year, setting, etc.)
Participants’ Characteristics	Sex, age, disabilities
Tele-Assessment Characteristics	Tele-test or tele-scale, delivery device types, synchronous and/or asynchronous, location, duration, results
Comparator Test Characteristics	Test or scale, location, duration, results
Primary Outcomes	Validity, inter-rater, intra-rater reliability, accuracy studies, minimal detectable change score, and measurement error study
Secondary Outcomes Measure	Other outcomes that the authors do not consider as primary clinical variables
Certainty of Evidence	Methods used

## Data Availability

The original contributions presented in this study are included in the article/Supplementary Material. Further inquiries can be directed to the corresponding author.

## Supplementary Materials

The following supporting information can be downloaded at: https://www.mdpi.com/article/10.3390/jcm14072311/s1, Supplementary File S1: Checklist Protocol Review [45]; Supplementary File S2: Query search.
