# Supplementary material for: Physiotherapist Online Assessment in Patients with Stroke: Protocol for a Systematic Review and Meta-Analysis
Source: J Clin Med. 2025 Mar 28;14(7):2311. doi: 10.3390/jcm14072311 (PMC11989669; doi:10.3390/jcm14072311)
Supplement: Supplementary file 1 [file jcm-14-02311-s001.zip › Supplementary File S2. Query search.pdf]

Supplementary File S2

DATABASE	QUERY SEARCH	RESULTS
Medline	Tele-assessment [Title/Abstract] OR Virtual-assessment [Title/Abstract] OR Remote-assessment [Title/Abstract] OR Outcome measures assessment [Title/Abstract] OR Digital physical therapy assessment "[Title/Abstract] OR Telerehabilitation [Title/Abstract] AND Stroke	35433
PeDro	Tele-assessment * AND Stroke<br>Virtual-assessment* AND Stroke<br>Remote-assessment* AND Stroke<br>Outcome measures assessment* AND Stroke<br>Digital physical therapy assessment* AND Stroke<br>Telerehabilitation* AND Stroke	496
Cochrane	Tele-assessment OR Virtual-assessment OR Remote-assessment OR Outcome measures assessment OR Digital physical therapy assessment OR Telerehabilitation AND Stroke	257
NICE	Tele-assessment OR Virtual-assessment OR Remote-assessment OR Outcome measures assessment OR Digital physical therapy assessment OR Telerehabilitation AND Stroke	183
